# A decade of research into classical swine fever marker vaccine CP7_E2alf (Suvaxyn^®^ CSF Marker): a review of vaccine properties

**DOI:** 10.1186/s13567-017-0457-y

**Published:** 2017-09-15

**Authors:** Sandra Blome, Kerstin Wernike, Ilona Reimann, Patricia König, Claudia Moß, Martin Beer

**Affiliations:** Institute of Diagnostic Virology, Friedrich-Loeffler-Institut, Suedufer 10, 17493 Greifswald-Insel Riems, Germany

## Abstract

Due to its impact on animal health and pig industry, classical swine fever (CSF) is still one of the most important viral diseases of pigs. To control the disease, safe and highly efficacious live attenuated vaccines exist for decades. However, until recently, the available live vaccines did not allow a serological marker concept that is essentially important to circumvent long-term trade restrictions. In 2014, a new live attenuated marker vaccine, Suvaxyn^®^ CSF Marker (Zoetis), was licensed by the European Medicines Agency. This vaccine is based on pestivirus chimera “CP7_E2alf” that carries the main immunogen of CSF virus “Alfort/187”, glycoprotein E2, in a bovine viral diarrhea virus type 1 backbone (“CP7”). This review summarizes the available data on design, safety, efficacy, marker diagnostics, and its possible integration into control strategies.

## Introduction

Classical swine fever (CSF) remains one of the most important viral diseases that affect sustainable pig production world-wide [14]. To control the disease in either endemic situations or in case of large, high-impact contingencies, safe and highly efficacious live attenuated vaccines exist for decades [50]. The underlying virus strains (e.g. the C-strain of CSFV or the guinea-pig exaltation negative GPE^−^-strain) were attenuated through serial passages in either animals (rabbits) or cell culture, and have been implemented as part of mandatory control programs that led to the eradication of CSF from several countries [19]. These vaccines are still in use in several Asian countries including China and were adapted to a bait format for oral immunization of wild boar [22, 23, 32]. While these vaccines have usually outstanding virtues in terms of onset and duration of immunity, the main drawback is the lack of a serological marker concept [49, 50] that would allow differentiating field strain infected from vaccinated animals (DIVA concept). In general, there are no legal obligations to use a certain type of vaccine for an emergency vaccination scenario. Yet, due to the trade restrictions that are imposed on pigs vaccinated with conventional live attenuated vaccines, only marker vaccines are considered a feasible option for immunization of domestic pigs [5]. Up to very recently, only E2 subunit marker vaccines were available on the market. These vaccines are safe but show drawbacks especially in terms of early protection and protection against vertical transmission [50]. Due to these problems, emergency vaccination was hardly implemented in domestic pigs (one exception being Romania). Several research groups have therefore focused on developing a next-generation marker vaccine candidate that would ideally answer all demands with regard to safety, efficacy, DIVA potential, and marketability. The ideal vaccine should fulfill all of the following requirements (postulated by Terpstra and Kroese [47]; modified by Dong and Chen [9] for marker vaccines): no short- or long-term side effects in vaccinated animals, genetic stability in both target and non-target species, stable and easy production under standard conditions, low costs of production, early onset of a robust and life-long immunity, efficacy against all virus variants, prevention of a carrier status, prevention of horizontal and vertical transmission, and availability of a highly sensitive and specific DIVA diagnostic test. It is obvious that meeting all demands is quite illusive. However, several promising attempts have been made. Among the concepts that have been pursued are different vector vaccines, recombinant attenuated vaccines (live and inactivated), subunit vaccines based on different expression systems, and RNA/DNA vaccines reviewed by [2, 5, 9]. It became evident that attenuated deletion vaccines and chimeric constructs showed high premises and the latter have been followed-up in the framework of two consecutive research projects funded by the European Union (EU). After comparative testing of candidate chimeras [4, 12] and thorough review of the available background data, “CP7_E2alf” was chosen as final candidate in the EU funded research project “Improve tools and strategies for the prevention and control of classical swine fever” (CSFV_goDIVA, KBBE-227003). The consortium partners tested the vaccine candidate “CP7_E2alf” for both intramuscular and oral application in accordance with the requirements for CSF vaccines that are provided by the European Pharmacopoiea (Ph. Eur., monograph 07/2008:0065) and the OIE (Office International des Epizooties, World Organisation for Animal Health) Manual of Diagnostic Tests and Vaccines for Terrestrial Animals (OIE Manual, chapter 2.8.3). Based on the results of these tests, a licensing dossier for the intramuscular vaccination was submitted to the European Medicines Agency (EMA). After review of the data and some supplementary trials, the vaccine candidate “CP7_E2alf” (Suvaxyn^®^ CSF Marker, Zoetis) was licensed as first live marker vaccine against classical swine fever. The presented review gives an overview on published or otherwise available data (see Table 1) with regard to construction, genetic stability, safety, efficacy, DIVA diagnostics, and strategy design.

**Table 1:** **Published studies on “CP7_E2alf” and their topics.**

Topic	Data covered by the article	References
Vaccine design and construction	Laboratory protocols for chimera design	[40]
	Construction of the chimera, sequence analysis, initial in vitro and in vivo tests	[39]
Genetic stability	Stability over cell culture passages, search for recombinants in co-infection studies, stability in vivo	[18]
Safety	Assessment of shedding through feces, urine and semen, dissemination	[10]
	Dissemination, onset of antibody responses, diagnostic tests	[48]
	Innocuousness and safety in target and non-target species	[26]
	Detection and dissemination of vaccine virus	[24]
Efficacy	Efficacy in the presence of MDA	[15]
	Efficacy in the presence of BVDV-1 antibodies, DIVA	[11]
	Efficacy in MDA negative piglets, intramuscular and oral vaccination with challenge at 14 dpv with CSFV “Koslov”	[29]
	Efficacy in piglets with MDA, intramuscular (3 weeks/6 weeks) and oral vaccination (6 weeks), challenge 14 dpv with CSFV “Koslov”	[13]
	Efficacy against different genotypes of CSFV, intramuscular and oral vaccination (domestic pigs and wild boar), challenge 14 dpv/21 dpv	[6]
	Efficacy after intramuscular vaccination and DIVA (comparative trial with different chimeras), challenge 7 and 14 dpv with CSFV “Koslov”	[12]
	Efficacy in piglets with C-strain derived MDA (5 weeks/8 weeks), challenge with CSFV “Koslov” 14 dpv	[38]
	Duration of immunity study, intramuscular and oral vaccination, challenge 6 months post vaccination with CSFV “Koslov”	[17]
	Efficacy after oral vaccination (comparative trial with different chimeras), challenge 14 and 21 dpv	[4]
	Onset of immunity and vaccine dose, efficacy study, genetic stability, intramuscular and oral vaccination	[27, 28]
	Efficacy (and safety) of oral immunization of wild boar	[25]
DIVA diagnostics	Development and validation of an E^rns^ -specific double-antigen ELISA	[34]
	Design and evaluation of an E^rns^ ELISA	[33]
	Evaluation of a discriminatory CSFV E^rns^ ELISA in an inter-laboratory trial	[37]
	Differentiation of CSFV infection and “CP7_E2alf” vaccination using a multiplex microsphere immunoassay	[53]
	Design of two E^rns^ antibody ELISAs	[1]
	Inter-laboratory comparison test of possible discriminatory assays	[45]
	Development of a RT-PCR system for vaccine/field virus discrimination (genetic DIVA)	[31]
	Development of a RT-PCR system for vaccine/field virus discrimination (genetic DIVA)	[27]
Field study	Oral vaccination of wild boar in faunistic hunting farms in Umbria, bait vaccination, comparative study in captive wild boar, vaccine stability	[16]
Supplemental studies	Kinetics of MDA upon intramuscular vaccination	[44]
	Cytokine and immunoglobulin isotype profiles	[42]
	Challenge 2 days after oral immunization, cytokine profiles	[41]

## Design and manufacturing

The pestivirus chimera “CP7_E2alf” was constructed based on the infectious cDNA clone of the cytopathogenic bovine viral diarrhea virus (BVDV) strain “CP7”. In this backbone, the E2 coding region was replaced with that of CSFV strain “Alfort/187” [39]. The parental BVDV was described by Corapi et al. [8], and generation of the cDNA construct is detailed in the respective publications by Meyers et al. [35, 36].

**Figure 1:**
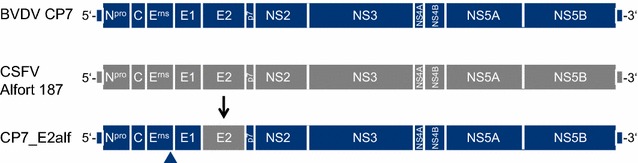
**Schematic representation of “CP7_E2alf” and its parental viruses BVDV “CP7” (represented in blue) and CSFV “Alfort/187” (grey).** The arrow indicates the position of the CSFV E2 (in grey) in the BVDV backbone (in blue). The arrowhead indicates the G^479^R mutation in BVDV-E^rns^, which is responsible for an efficient virus growth in porcine cells.

The subsequent steps for the generation of the chimera are detailed by Reimann et al. [39]. In brief, an E2-deleted cDNA construct was generated and the E2 encoding region of CSFV “Alfort/187” was inserted by a classical cloning procedure using singular restriction sites (see Figure 1). The final vaccine virus “CP7_E2alf” was obtained through transfection (electroporation) of in vitro-transcribed RNA from the linearized chimeric plasmid “pA/CP7_Ealf” into porcine kidney and bovine esophageal cells. It was demonstrated that with the acquisition of CSFV E2, the chimera behaves like a CSFV strain and replicates efficiently in porcine cell lines. The 11th passage on porcine cells was used for initial evaluation in vivo and for sequence generation.

The manufactured vaccine is now grown in porcine kidney cells (SK cells) in either roller bottles or bioreactors. The final bulk antigen is stabilized with a commercial stabilizer and formulated in a lyophilized way. The summary of product characteristics can be found at the EMAs webpage: http://www.ema.europa.eu/docs/en_GB/document_library/EPAR_-_Product_Information/veterinary/002757/WC500185867.pdf (visited August 7th 2016).

In brief: a chimeric pestivirus was constructed based on the infectious cDNA clone of BVDV “CP7″. In this backbone, the E2 was exchanged with that of CSFV “Alfort/187”. The resulting virus “CP7_E2alf” replicates mainly in porcine cells.

## Genetic stability

While safety and efficacy are crucial parameters for an emergency vaccine, it is also essential to demonstrate that a genetically engineered RNA virus is genetically stable, i.e. does at least not show higher mutational rates as its parental viruses.

To generate reliable sequence data for construct characterization and comparison of the 1^st^ and 11^th^ passage of “CP7_E2alf”, PCR products spanning different fragments of the genome region encoding the structural proteins were cloned into plasmids (pGEM-T Easy Vector System; Promega). A minimum of eight clones per fragment taken from individual PCR runs were sequenced along with direct sequencing of the respective RT-PCR products [39]. Under these settings, amino acid (aa) changes were observed in the regions encoding E^rns^ at positions H^450^R, and G^479^R, E1 at position L^686^H, E2 at positions M^783^T, S ^973^P, R^1023^H, and V^1035^E. Of these variations, the G^479^R exchange is of special interest, as it plays a major role for the interaction with membrane-associated heparan sulfate and thus cell culture adaptation [21]. Only this exchange was observed when direct sequencing of RT-PCR products was performed. This implies that multitude of the above mentioned changes are only found in a part of the viral quasi-species and that they are not reflected in the consensus sequence. Later on, a sequence comparison was undertaken between the already mentioned 11^th^ passage of “CP7_E2alf” and the first GLP (good laboratory practice) produced batch [28]. In general, the DNA sequence of the 11th passage was confirmed also in the GLP batch. However, a total of 10 mutations was identified. Two mutations concerned the 5′-non translated region, and non synonymous substitutions were observed in each the capsid protein C (D^136^ N), E^rns^ (L^478^F), E2 (S^981^P), p7 (Y^1071^C), NS2 (V^1561^L) and NS4B (AM^2457^V), and two substitutions were found in the NS5B (S^3341^I, T^3885^K) encoding region. It is obvious that only one mutation concerned the immunogen E2, and it does however not affect the known B- or T-cell epitopes. The other changes in the backbone cannot influence the induction of neutralizing antibodies. Judging on the genetic stability, it should be kept in mind that the variability of the vaccine virus was narrowed down upon establishment of the master seed virus through end-point dilution of the pre-master seed virus.

Stability of the final vaccine formulation was addressed in in vitro and in vivo studies reported by Goller et al. [18]. In brief, stability of the vaccine virus, its parental viruses, and two prototype field strains of CSFV and BVDV was assessed upon cell culture passaging. Moreover, co-infection experiments were attempted to address recombination, and an in vivo study was followed up by next-generation sequencing. Compared to the above mentioned GLP stock, the formulated vaccine carried only one additional substitution in the E^rns^ protein gene. Ten cell culture passages did not lead to additional changes in the consensus full-length sequence. On quasispecies level (high frequency variants), no substitutions were observed in the first five passages. In passage 10, five high frequency variants were observed, all in the backbone sequence outside the structural protein genes. Moreover, no recombination events could be demonstrated. The comparison of the vaccine virus and the virus in a tonsil from an animal trial revealed one substitution in the NS5B region of “CP7_E2alf”. Due to the low viral load, the latter comparison was only possible based on overlapping PCR products [18].

In brief: “CP7_E2alf” proved to be highly stable both in vitro and in vivo. No indications exist for recombination events or an increase in mutational events compared to its parental viruses.

## Safety and vaccine virus distribution

Following the design and initial testing of “CP7_E2alf”, innocuousness for target and non-target species was investigated. For the assessment of the latter, high titres of “CP7_E2alf” were applied orally to calves, goat kids, sheep lambs, and young rabbits. Additional animals were left as sentinels for transmission. None of the animals seroconverted and vaccine virus was not found in any of the samples taken from both vaccinees and sentinels [26]. In the target species, innocuousness, shedding and transmission were investigated in the framework of several studies with oral and intramuscular vaccination of both, domestic pigs and European wild boar. Up to now, none of the published or elsewhere reported studies reported local or systemic side effects, and in studies with sentinel pigs, vaccine virus was never transmitted [26]. The studies on the effect of maternally derived BVDV antibodies (MDA) showed safety for piglets from an age of 3 weeks and unpublished studies in pregnant sows showed that the vaccine virus does not cross the placental barrier.

With regard to virus distribution, König et al. [24] studied detectability and distribution of “CP7_E2alf” upon single intramuscular vaccination over a period of 42 days. Furthermore, shedding and transmission of vaccine virus was assessed. It was demonstrated that the virus was not transmitted to commingled sentinels, and detectability by virus isolation and real-time RT-PCR was very short-lived (in the vast majority of cases < 1 week) in blood samples. With regard to virus and genome detection in organ samples, live virus was only detected in one tonsil sample at 6 dpv, but genome was detected consistently in tonsils up to 42 dpv and in lymphatic organs in the 2 weeks following vaccination. In a later study, it was shown that vaccine virus genome was detectable in tonsils for up to 63 days after oral immunization [48]. The above mentioned results are in line with further studies where detection in blood samples after oral or intramuscular vaccination was absent [39, 48] or very short-lived and low [4, 10].

Supplementary studies were done to assess the presence of the vaccine chimera in faeces, urine, and organs of the male reproductive tract [10]. To this end, twelve adult boars were vaccinated with a tenfold vaccine dose of “CP7_E2alf” under controlled laboratory conditions. While viral genome was detectable in several samples from lymphatic tissues, infectious virus was only re-isolated at 4 days post vaccination in one tonsil sample and one parotid lymph node. Sporadic detection of viral RNA at a very low level occurred in some replicates of liver, lung, bone marrow, and salivary gland samples. In contrast, viral genome was not detected in any sample from reproductive organs and accessory sex glands, in faeces, urine or bile. Generally, the tonsil seems to be the primary replication site both after oral and intramuscular vaccination [10, 24, 48].

In brief: “CP7_E2alf” proved to be innocuous and safe in target and non-target species. Limited replication occurs primarily in the tonsils and is detectable there over several weeks mainly through the use of real-time RT-PCR. The vaccine virus is not transmitted or shed through urine, faeces or semen.

## Efficacy

In the framework of the initial studies reported by Reimann et al. [39], intramuscular inoculation of 1 × 10^7^ tissue culture infectious doses 50% (TCID_50_) of “CP7_E2alf” conferred full protection against challenge infection 28 dpv with the highly virulent CSFV strain “Eystrup”.

Efficacy for oral immunization of wild boar was first addressed in the study reported by König et al. [25]. It was shown that orally vaccinated wild boar were fully protected against highly virulent challenge with CSFV “Koslov” also at 28 dpv.

Later on, it was demonstrated that full clinical protection against highly virulent challenge with CSFV “Koslov” was conferred 1 week after intramuscular and 2 weeks after oral vaccination [28]. Moreover, protection was also demonstrated using diluted vaccine preparations (down to 2 × 10^4.25^ TCID_50_), and challenge after 28 days [28]. These results were confirmed in the comparative studies that led to the choice of “CP7_E2alf” as vaccine candidate for licensing. Protection against clinical disease, mortality, and virus transmission was demonstrated for a challenge with a highly virulent CSFV strain (“Koslov”) seven or 14 days after single intramuscular vaccination. In the oral immunization part [4], full protection was demonstrated 21 days post challenge (dpc). With challenge at 14 dpc, full clinical protection was conferred, but transmission to contact controls could not be excluded.

Supplementary studies with very early challenge showed partial protection as early as 2 dpv [41]. In this study, animals were challenged with the moderately virulent genotype 2.3 CSFV strain “Bas-Rhin” 2 days after oral immunization with either C-strain vaccine or “CP7_E2alf”.

Lately, an additional pre-registration efficacy study with MDA negative piglets was published by Lévai et al. [29]. This study comprised both oral and intramuscular inoculation with CSFV “Koslov” challenge at 14 dpv. Lévai et al. reported full protection and thus compliance with the respective monograph of the European Pharmacopoeia upon intramuscular vaccination but only partial protection upon oral immunization. The orally vaccinated animals showed mild symptoms in the majority of cases but one animal had to be euthanized for animal welfare reasons. This animal had not seroconverted upon vaccination. Virus detection was short-lived in the affected animals except for the animal that had to be euthanized. Taken together, oral vaccination seemed to be at its limits under these conditions with challenge 14 days and it should be kept in mind that oral administration is rather error prone.

As part of the licensing procedure, duration of immunity had to be investigated. As mentioned in the OIE Manual and the Phr. Eur., duration of immunity after vaccination against CSF shall not be less than 6 months. This has to be demonstrated in at least 10 vaccinated pigs. A corresponding study [17] was undertaken for both intramuscular and oral vaccination with “CP7_E2alf”. Within the study, it was demonstrated that the duration of immunity after single intramuscular vaccination was at least 6 months and thus, the vaccine complied with the requirements. In the trial part with oral vaccination, one animal did not respond to vaccination and succumbed to challenge infection. Apart from this non-responder, all animals were also completely protected upon challenge 6 months after vaccination.

To address the request for an efficacy and cross-protection against all strains and types of field viruses, an efficacy trial with intramuscular and oral vaccination was carried out using challenge strains of more recent genotypes, namely 2.1 (CSF1047, Israel, 2009) and 2.3 (CSFV1045, Germany, 2009) along with CSFV “Koslov” of genotype 1. The study demonstrated that solid protection was achieved against all employed genotypes and thus, broad applicability under relevant field conditions can be assumed [6].

For the risk assessment towards (emergency) vaccination strategies that include breeding animals, the impact of maternally derived antibodies (MDA) is a crucial parameter. For “CP7_E2alf”, one study was so far published that addresses “CP7_E2alf” derived MDA [13]. Two other studies [15, 38] mimicked the situation in endemically infected countries with previous vaccination using live attenuated vaccines. In the study published by Eble et al. [13], piglets with and without MDA were enrolled in a vaccination-challenge-trial at the age of 3 and 6 weeks for intramuscular vaccination and 6 weeks for oral vaccination. Taken together, the results suggest that in this age group of animals (very young piglets) “CP7_E2alf” is an effective tool on population level but shows slightly reduced efficacy.

In the study reported by Rangelova et al. [38], pregnant sows were vaccinated once with C-strain 4 weeks before farrowing. The offspring was later on vaccinated at the age of 5 and 8 weeks and challenged 2 weeks later with the highly virulent CSFV strain “Koslov”. Maternal immunity (C-strain) alone reduced mortality, however, additional vaccination with “CP7_E2alf” provided clinical protection upon challenge and virus isolation was not possible from the animals post challenge. Only some minor clinical signs such as short fever, mild leukopenia, and depression over a few days were observed. Very recently, Farsang et al. [15] reported on a study with CSFV “Thiverval” vaccinated sows. Six weeks after farrowing, groups of piglets were either orally or intramuscularly vaccinated with “CP7_E2alf”. Two weeks later, the animals were challenged with highly virulent CSFV strain. The negative influence of MDA was confirmed, especially for the orally vaccinated animals. However, protection against CSF induced mortality and significant reduction of clinical signs was observed.

Very recently, efficacy of intramuscular vaccination was also confirmed in animals with pre-existing BVDV-1 antibodies [11]. The study with C-strain “Riems” as comparator involved intramuscular vaccination of pre-exposed and naïve animals with either “CP7_E2alf” or C-strain “Riems” and oro-nasal challenge with CSFV “Koslov” 7 days later. Both C-strain “Riems” and “CP7_E2alf” were able to confer full protection against highly virulent CSFV challenge infection. However, interference was seen with serological DIVA diagnostics accompanying the vaccination with CP7_E2alf (see below).

In brief: “CP7_E2alf” showed repeatedly that protection is conferred within 1 week post intramuscular vaccination or even less, and the immunity lasts for at least 6 months. Oral vaccination and vaccination of MDA positive or very young piglets can slightly reduce the efficacy.

## Diva

### Genetic DIVA

Live attenuated vaccine strains such as the C-strain or “CP7_E2alf” show a very limited replication even in the target host [24] and thus, re-isolation of vaccine virus is a rare event. However, highly sensitive detection techniques such as real-time RT-PCR can lead to vaccine virus detection in blood and organs. This was repeatedly seen in animal trials and under field conditions where wild boar received oral C-strain vaccination [3]. To rapidly differentiate these detections from field virus infection (genetic DIVA), specific real-time RT-PCR systems have been developed for different C-strain variants and marker vaccine CP7_E2alf [20, 27, 30, 31].

### Serology

The serological marker system of “CP7_E2alf” is based on the detection of CSFV-specific E^rns^ antibodies. Animals vaccinated with “CP7_E2alf” will carry CSFV E2 but not CSFV E^rns^ antibodies while field virus infected animals will also show CSFV E^rns^ responses. At present, two CSFV E^rns^ ELISAs are commercially available. One is the PrioCHECK CSFV E^rns^ (Thermofisher, former Prionics), the other the pigtype CSF Marker (Qiagen).

In general, there are some biological issues that influence the performance of the discriminatory assays and limit their optimization. If for example solid and almost sterile immunity/protection is achieved, the lack of challenge virus replication might also lead to a missing E^rns^ response. In this case, a negative result is not caused by a lack of sensitivity but represents the true status of the sample [4]. Another problem is the lack of knowledge regarding the onset and kinetics of the E^rns^ antibody responses under field conditions. Taken together, there is still room for improvement, and the test systems should target populations/herds rather than individual animals.

In a first inter-laboratory assessment of CSFV antibody detection assays that could be used for the differentiation of infected from vaccinated animals, the E2 antibody ELISAs showed highest sensitivity and specificity. Under these ringtrial conditions, the PrioCHECK CSFV E^rns^ antibody ELISA showed problems with sensitivity and cross-reactions with other pestiviruses [45]. It has to be noted that successive batches were later on tested in the framework of different vaccine and infection trials [6, 17, 48]. In these studies, the sensitivity was much higher. Cross-reactions with other pestiviruses were however confirmed, e.g. in the study on the efficacy of the vaccine in BVDV-1 antibody positive animals [11].

In the comparative study published by Eble et al. [12], all “CP7_E2alf” vaccinated animals became seropositive upon challenge, but sometimes as late as 35 or 49 dpc. However, a majority of the negative-vaccinated samples showed higher inhibition percentages than completely negative samples and, some were very close to the cut-off value of the test. It was calculated that about 5% of samples tested in a comparable population with normal distribution could exceed the 40% cut-off value of the PrioCHECK CSFV E^rns^ ELISA and would therefore score false positive. Based on the same assumptions, false positives in a negative (unvaccinated) population would be very unlikely (< 0.001% of samples tested in a comparable population). Eble et al. observed also weaknesses with regard to cross-reactivities with BVDV-2 and (to a lesser extent) Border disease virus (BDV) antibodies.

Recently, another ringtrial was conducted among five international partners to review the current version of the PrioCHECK CSFV E^rns^ [37]. Based on 530 serum samples and a set of country-specific negative sera, a sensitivity of 90–98% with sera from CSFV infected animals, and a specificity of 89–96% for sera from vaccinated animals was calculated. Classical swine fever infection in vaccinated animals was detected with a sensitivity of 82–94%. A considerable cross-reactivity was still observed with both BVDV and BDV strains, and multiple vaccination and limited sample quality reduced specificity.

Recently, data on the evaluation of the Erns—specific double-antigen ELISA (pigtype CSFV E^rns^ Ab, Qiagen) were published [34]. It was reported that the overall sensitivity to detect E^rns^ -specific antibodies against CSFV isolates of different genotypes was 93.5% combined with a specificity of 99.7%. In combination with “CP7_E2alf”, the novel ELISA showed a sensitivity of 90.2% and a specificity of 93.8%. Cross-reactivity with antibodies against ruminant pestiviruses was observed. In the framework of the evaluation, it was confirmed that false positive reactions are rarely found in both available Erns antibody ELISA systems.

To further optimize the discriminatory systems and to allow the combination of diagnostic tests, additional approaches are currently under development based on either ELISA or Luminex technology [1, 53]. In addition, alternative strategies such as the recently reported discrimination within epitope specific antibody populations [7] could be considered.

Recently, yet another CSFV E^rns^ antibody ELISA was reported that uses *Pichia pastoris* expressed E^rns^ in an indirect ELISA format. The available validation data point to good overall sensitivity (95%) and specificity (97%). At least under the reported conditions, this indirect ELISA was superior to the commercial PrioCHECK CSFV E^rns^ ELISA [33].

In brief: suitable tools are available for both genetic and serological DIVA. The latter has still some room for improvements and has to be embedded in a concerted control strategy.

## Immunology

The onset of detectable E2 specific antibodies was reported between 11 and 15 days after oral immunization [48] and in this case, antibodies were still present 98 dpv. This experience was quite in line with the above mentioned study by König et al. [24], where neutralizing antibodies were detectable from 8 dpv in “CP7_E2alf” vaccinated animals, and first E2 antibodies were detected by ELISA from 11 dpv (usually between 14 and 21 dpv). After oral immunization, antibodies were detectable within 2 to 3 weeks post vaccination [25]. It seems that compared to C-strain “Riems”, serological responses upon oronasal vaccination can be slightly delayed and reduced with high variability among animals [48]. This does however not affect efficacy. In the duration of immunity studies, antibodies persisted for at least 6 months [17], and personal observation show that antibodies persist for several years in “CP7_E2alf” vaccinated captive wild boar.

In terms of MDA kinetics, a separate study group was monitored for the decay of MDA in the study published by Eble et al. [13]. Based on neutralization test results, a half-life of 13.8 days was estimated for “CP7_E2alf”-derived MDA starting from 20 to 640 ND_50_ at sampling 1 week after birth. These estimates are in line with C-strain data, where the half-life was reported to be approximately 12 days [46]. The corresponding “CP7_E2alf” vaccinated sows had a maximum Neutralizing Peroxidase Linked Assay (NPLA) titer of 80 to 1280 ND_50_. Recently, MDA kinetics were also investigated after single vaccination of pregnant sows 21 days before farrowing [44]. The E2 ELISA reactivities showed an almost linear decrease over 10 weeks after which all piglets were tested negative in the ELISA again. No problems were observed in DIVA assays (Erns antibodies) when heat-inactivated sera were used.

Renson et al. [41] showed that production of CSFV-specific IgG1 and neutralizing antibodies without challenge was lower with “CP7_E2alf” vaccination than with C-strain vaccination. This could suggest a difference in the balance of adaptive immune responses despite their comparable induction of protection. Further investigations with regard to cytokine and immunoglobulin isotype profiles showed significant reduction of proinflammatory cytokine levels upon challenge, especially TNF-α and IL-6, and suggested an important role of cell-mediated immunity in both short- and long-term protection against CSFV [42]. However, IgA production also revealed stimulation of mucosal immunity, especially upon oral immunization.

In brief: usually, “CP7_E2alf” induces antibodies within the first 2 weeks post vaccination and the antibodies persist for at least 6 months.

## Field study

Due to legal restrictions (prophylactic vaccination is prohibited within the EU), field studies were difficult to implement. However, it was possible to carry out a small study on oral immunization in two so-called “faunistic hunting farms” in Umbria, Italy [16]. For this purpose, the oral vaccine was formulated in baits that are regularly used for C-strain vaccination. One campaign was carried out with single vaccination, the other with the routinely employed double vaccination. Bait uptake, vaccine virus detection and antibody responses were investigated. Bait uptake ranged from 63.7 to 98.7% whereas antibody prevalences of 33.3 to 35.1% were determined. An impact of sample quality on the discriminatory CSFV E^rns^ antibody ELISA (PrioCHECK CSFV Erns, Thermofisher, former Prionics) was observed with a total of 85% of animals scoring correctly negative.

In brief: a first field trial gave promising results using the bait formulation routinely used for C-strain vaccination.

## Strategy design and further needs for the implementation

With the licensing of “CP7_E2alf”, another tool of CSF control has become available and warrants revisiting the option of emergency vaccination of both domestic pigs and wild boar. Marker vaccines can only be part of a concerted control action and are no substitute for sanitary measures [51]. Such measures also need inclusion of all relevant stakeholders. It is of particular importance to design testing schemes and exit scenarios beforehand and to discuss ways of trade. Testing schemes should take the obvious limitations in DIVA diagnostics into consideration and the latter should be reflected in the sample size. It should also be discussed to put more emphasis on the detection of the field virus itself and genetic DIVA. Combination of detection methods (different serology assays together with genetic DIVA tests), and results analysis on the farm or region level will allow reliable strategies implementing the novel CSFV marker vaccine. As a global reference, the terrestrial Manual of the OIE should consider to include the progress described in this overview. So far, it describes subunit DIVA vaccines with their limits as described here above. We suggest completing Chapter 2.8.3 on Classical swine fever with the findings on “CP7_E2alf”. This would lead to increased awareness for more efficient control of CSF.

In the recent past, it was demonstrated that oral vaccination of CSF affected wild boar populations can help to control the disease and prevent introduction into the domestic pig population [22, 52]. So far, vaccination of wild boar was limited to the use of C-strain based bait vaccines, and the serosurveillance was not able to differentiate immunity upon field virus infection from vaccine induced antibodies. Thus, it was not easy to determine the end of a vaccination campaign. Live marker vaccines would in general be able to overcome these problems [43] and could be implemented in the same manner as C-strain vaccines. With regard to the use in wild boar, licensing of “CP7_E2alf” is still pending and should be followed up. While the majority of the above mentioned studies was done for both intramuscular and oral vaccination, questions on bait formulation remain and a licensing request has to be submitted to EMA. Even if the performance of the DIVA assay shows weaknesses when using wild boar samples, benefit should still be high on population level.

In brief: “CP7_E2alf” is a new instrument in the tool-box of CSF control. It can be used to revisit emergency vaccination scenarios.

## Abbreviations

aa: amino acid
BDV: Border disease virus
BVDV: bovine viral diarrhea virus
CSF: classical swine fever
CSFV: classical swine fever virus
DIVA: differentiating field strain infected from vaccinated animals
dpc: days post challenge
dpv: days post vaccination
EMA: European Medicines Agency
EU: European Union
GLP: good laboratory practice
MDA: maternally derived antibodies
OIE: Office International des Epizooties, World Organisation for Animal Health
Ph. Eur.: European Pharmacopoiea
TCID_50_: tissue culture infectious doses 50%
